# Memristive Hodgkin-Huxley Spiking Neuron Model for Reproducing Neuron Behaviors

**DOI:** 10.3389/fnins.2021.730566

**Published:** 2021-09-23

**Authors:** Xiaoyan Fang, Shukai Duan, Lidan Wang

**Affiliations:** ^1^School of Electronic and Information Engineering, Southwest University, Chongqing, China; ^2^College of Artificial Intelligence, Southwest University, Chongqing, China; ^3^Brain-Inspired Computing and Intelligent Control of Chongqing Key Lab, Chongqing, China; ^4^National and Local Joint Engineering Laboratory of Intelligent Transmission and Control Technology, Chongqing, China; ^5^Chongqing Brain Science Collaborative Innovation Center, Chongqing, China

**Keywords:** HH, MHH, memristor, neuron, spiking

## Abstract

The Hodgkin-Huxley (HH) spiking neuron model reproduces the dynamic characteristics of the neuron by mimicking the action potential, ionic channels, and spiking behaviors. The memristor is a nonlinear device with variable resistance. In this paper, the memristor is introduced to the HH spiking model, and the memristive Hodgkin-Huxley spiking neuron model (MHH) is presented. We experimentally compare the HH spiking model and the MHH spiking model by applying different stimuli. First, the individual current pulse is injected into the HH and MHH spiking models. The comparison between action potentials, current densities, and conductances is carried out. Second, the reverse single pulse stimulus and a series of pulse stimuli are applied to the two models. The effects of current density and action time on the production of the action potential are analyzed. Finally, the sinusoidal current stimulus acts on the two models. The various spiking behaviors are realized by adjusting the frequency of the sinusoidal stimulus. We experimentally demonstrate that the MHH spiking model generates more action potential than the HH spiking model and takes a short time to change the memductance. The reverse stimulus cannot activate the action potential in both models. The MHH spiking model performs smoother waveforms and a faster speed to return to the resting potential. The larger the external stimulus, the faster action potential generated, and the more noticeable change in conductances. Meanwhile, the MHH spiking model shows the various spiking patterns of neurons.

## 1. Introduction

Neurons with highly nonlinear characteristics act as the basic functional unit of receiving and propagating signals. The whole procedure of processing signals in the nerve system needs the cooperation of neurons. Some theoretical knowledge and research methods are beneficial to unveil the mechanism of information propagation in neurons. Italian scientist Camillo Golgi worked on the nervous system structure and earned the Nobel Prize for physiology and medicine in 1906 (Dröscher, 1998). In 1998, Ramon y Cajal pointed out that the neurons without directly connecting each other in the nerve system (Raviola and Mazzarello, 2011). To replicate the functions and mechanisms of neurons, we urgently need to construct the biophysical model. A variety of neuron models are emerging, and the Hodgkin-Huxley (HH) spiking neuron model is the original (Hodgkin and Huxley, 1989). Stochastic Hodgkin-Huxley Neuron Systems with the NEF is helpful to study neuron sensitivity (Chen and Li, 2010). The Hodgkin-Huxley Model with automatic parameter estimation is applied to the neuromimetic chips (Buhry et al., 2011). The space-clamped Hodgkin-Huxley model effectively inhibits the production of spikes under the injection of the noisy synaptic input (Tuckwell and Ditlevsen, 2016). The Langevin is combined with the Hodgkin-Huxley system performs accurate interspike interval (ISI) and realizes the accuracy minimal loss (Pu and Thomas, 2020). The Berger-Levy theory is introduced to the Hodgkin-Huxley model, demonstrate that the information communication between neurons is related to the presynaptic firing rate and the synchronization (Ghavami et al., 2018).

The memristor with the non-volatility and variable resistance characteristics is regarded as the fourth passive circuit element. Therefore, it becomes a hot topic in neural computing (Le et al., 2015), learning and memorizing (Sayyaparaju et al., 2018), micro-circuitry design (Berdan et al., 2014), biological synapse (Mandal and Saha, 2016), and neuron modeling (Maheshwar et al., 2014), and so on. The synaptic plasticity of biological neuronal systems can be realized by memristors and memristive crossbar in 3-D architecture to mimic the human brain (Truong et al., 2016). The memristor with hysteresis and memory characteristics is the most promising candidate for establishing the brain-like neuromorphic system (Mokhtar et al., 2017). The key features of biological neurons and synapses can be mimicked by memristors (Berdan et al., 2016; Mandal and Saha, 2016). The ion motion in neurons is represented by the electrical conductance change of a memristor (Xia and Yang, 2019). A memristor is used as a two-terminal resistor with memory (Chua, 1971; Strukov et al., 2008) performs well in storing information according to the physical laws (Yang et al., 2013). The memristor entirely avoids the data transformation bottleneck between the memory and computation (Li and Wang, 2019). The memristor crossbar array can be used to integrate the co-processor chip, which will realize machine learning algorithms and neuromorphic computing (James, 2019).

This work elaborates on the construction of the memristive Hodgkin-Huxley spiking neuron model. The mathematical expressions and the circuit of the HH spiking model are presented and analyzed in sections 2, 3. Section 4 describes the MHH spiking model and discusses the memristors used to mimic the ion channels. The comparison between two models under the different stimuli is conducted in section 5. Section 6 is the conclusion of the paper.

## 2. The Hodgkin-Huxley (HH) Spiking Neuron Model

The neuron cell membrane is a voltage-gated ion channel, which has high selectivity for the permeability of external and internal ions in body fluid. Only one type of ion can pass through specific channels. There involves four ionic components, sodium, potassium, calcium, and chloride. The transmembrane current depends on the rapid inward current caused by sodium and the slow outward current caused by potassium (Häusser, 2000). The ion concentration difference inside and outside of the cell is the primary driving force of neural activities. When the sodium channels are opened, the high concentration sodium flows from extracellular to intracellular, the depolarization is produced, the action potential is generated. And then, the sodium channels are closed, and the potassium channels are opened, the potassium permeates from intracellular to extracellular, the repolarization is performed. Finally, the membrane potential undergoes a hyperpolarization phase, the membrane potential shifts back to the resting potential. The above process is the generation mechanism of the action potential in a neuron.

**Figure 1:**
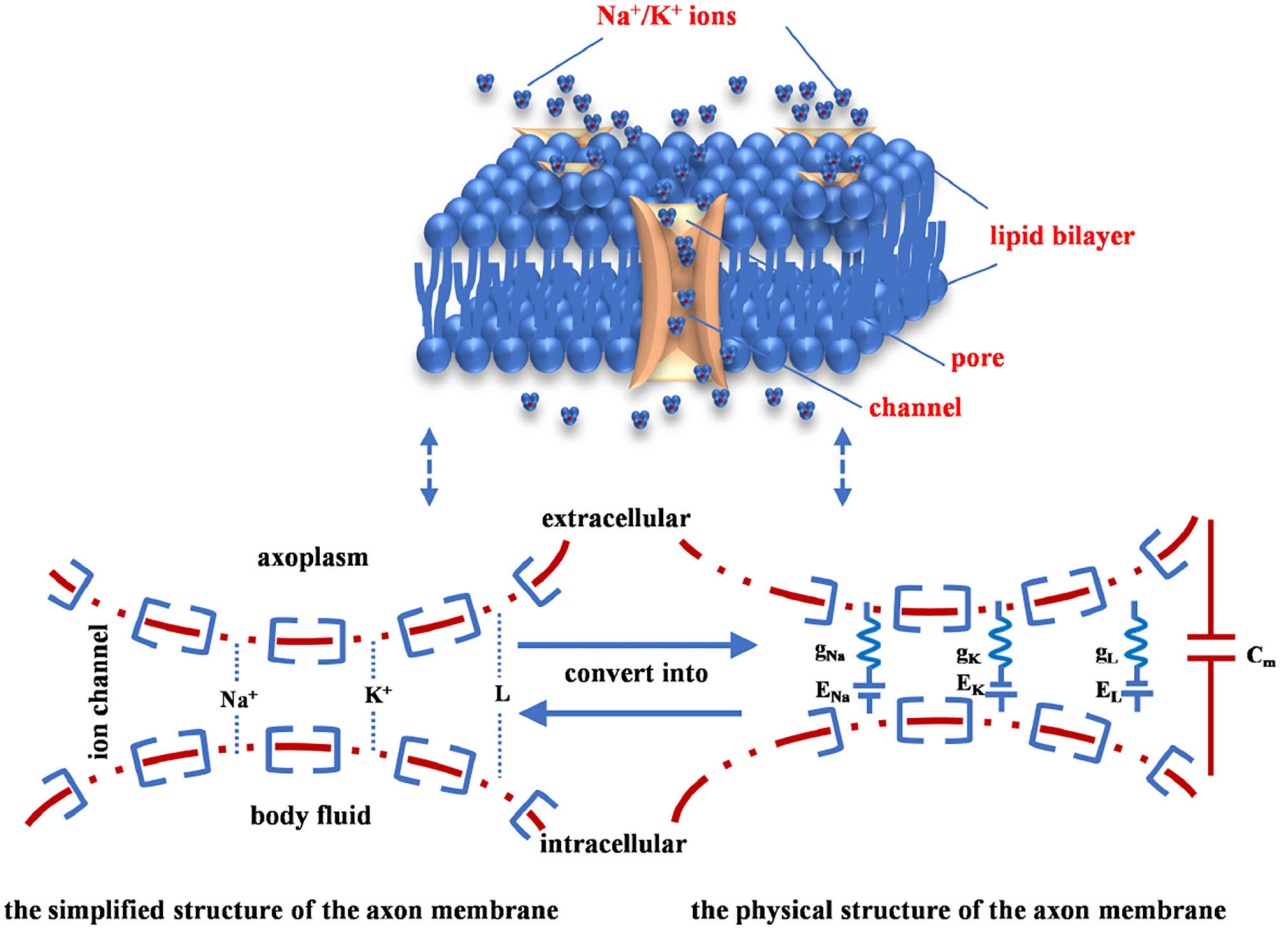
The voltage-gated channel of the axon cell membrane.

The inside of the axon membrane is full of ionic fluids (cytoplasm), the outside of the axon membrane is filled with body fluids. The fluids (conductor) of intracellular and extracellular are separated by the axon membrane (insulator). When an insulator separates two conductors, the capacitor emerges to model the charge storage capacity. The part of the axon membrane without ion channels is equivalent to a capacitor (*C*_*m*_). The axon membrane of the neuron consists of the lipid bilayer, the membrane protein, and ion channels (the upper image in Figure 1). The sodium ion channel is represented by a nonlinear conductance (*g*_*Na*_), the potassium ion channel is denoted by a nonlinear conductance (*g*_*K*_), and other ion channels are described as a linear conductance (*g*_*L*_) (Beck et al., 2020). When the neuron is in the resting state, a potential difference is caused by the ionic concentration between the intracellular and extracellular fluids. The potential difference is called the equilibrium potential of each ion (*E*_*Na*_, *E*_*K*_, and *E*_*L*_), which is equivalent to a driving power supply (the lower image in Figure 1).

When the neuron is in the resting state, there is a resting potential. Here, we choose *v*_*rest*_ = −65 mV as the resting potential in experiments (Hodgkin and Huxley, 1952). The *V*_*m*_ denotes the membrane potential, *E*_*Na*_ (50 mV), *E*_*K*_ (−70 mV), and *E*_*L*_ (−50 mV) represent the Nernst equilibrium potentials. When the potassium current passes through the potassium channel, the potassium current is proportional to the difference between the membrane potential and *E*_*K*_ (Hodgkin and Huxley, 1989; Börgers, 2017):


(1) $$\begin{array}{l} I_{K}=g_{K}\left(V_{m}-E_{K}\right) \end{array}$$

Here, *g*_*K*_ is the potassium conductance, (*V*_*m*_-*E*_*K*_) is the potassium driving force. The sodium current and the leaky current are described as:


(2) $$\begin{array}{l} I_{Na}=g_{Na}\left(V_{m}-E_{N}a\right) \end{array}$$

(3) $$\begin{array}{l} I_{L}=g_{L}\left(V_{m}-E_{L}\right) \end{array}$$

The ion channels are sensitive to membrane potential, which control the open and close states of channels.

In the Hodgkin-Huxley spiking model, the conductance value of each ion channel is decided by the gate-controlled variables m, n, h, and 0 ≤ *m* ≤ 1, 0 ≤ *n* ≤ 1, 0 ≤ *h* ≤ 1. The potassium channel depends on four active gate variables (n). The sodium channel is controlled by three active gate variables (m) and one inactive gate variable (h). The potassium conductance, the sodium conductance, and the leaky conductance are described as:


(4) $$\begin{array}{l} g_{K}=g_{Kmax}n^{4} \end{array}$$

(5) $$\begin{array}{l} g_{Na}=g_{Namax}m^{3}h \end{array}$$

(6) $$\begin{array}{l} g_{L}=g_{Lmax} \end{array}$$

Here, *g*_*Kmax*_, *g*_*Namax*_, and *g*_*Lmax*_ denote the maximum values of potassium, sodium, and leaky conductances, accordingly. Their values are 36, 120, 0.3 *Ohm*^−1^*cm*^−2^ (Hodgkin and Huxley, 1952, 1989). The expressions of gate-controlled variables of ion channels are written as follows:


(7) $$\begin{array}{l} dm/dt=1/\tau_{m}\left(m_{\infty }-m\right) \end{array}$$

(8) $$\begin{array}{l} dn/dt=1/\tau_{n}\left(n_{\infty }-n\right) \end{array}$$

(9) $$\begin{array}{l} dh/dt=1/\tau_{h}\left(h_{\infty }-h\right) \end{array}$$

The time constants τ_*m*_, τ_*n*_, and τ_*h*_ change with m, n, and h, accordingly. The transition rate α characterizes the ion channels change from the close state to the open state. The transition rate β indicates the ion channels vary from the open state to the close state. *m*_∞_, *n*_∞_, and *h*_∞_ are the steady-state values of the gate variables m, n, and h, accordingly (Saïgai et al., 2011). They are all the functions of the membrane potential. Their expressions are:


(10) $$\begin{array}{l} m_{\infty }=\alpha_{m}/\left(\alpha_{m}+\beta_{m}\right) \end{array}$$

(11) $$\begin{array}{l} n_{\infty }=\alpha_{n}/\left(\alpha_{n}+\beta_{n}\right) \end{array}$$

(12) $$\begin{array}{l} h_{\infty }=\alpha_{h}/\left(\alpha_{h}+\beta_{h}\right) \end{array}$$

(13) $$\begin{array}{l} \tau_{m}=1/\left(\alpha_{m}+\beta_{m}\right) \end{array}$$

(14) $$\begin{array}{l} \tau_{n}=1/\left(\alpha_{n}+\beta_{n}\right) \end{array}$$

(15) $$\begin{array}{l} \tau_{h}=1/\left(\alpha_{h}+\beta_{h}\right) \end{array}$$

(16) $$\begin{array}{l} \alpha_{m}=\varphi \left(2.5-0.1\left(V_{m}-V_{rest}\right)\right)/\left(e^{\left(2.5-0.1\left(V_{m}-V_{rest}\right)\right)}-1\right) \end{array}$$

(17) $$\begin{array}{l} \alpha_{n}=\varphi \left(0.1-0.01\left(V_{m}-V_{rest}\right)\right)/\left(e^{\left(1-0.1\left(V_{m}-V_{rest}\right)\right)}-1\right) \end{array}$$

(18) $$\begin{array}{l} \alpha_{h}=0.07\varphi e^{\left(-\left(V_{m}-V_{rest}\right)\right)/20} \end{array}$$

(19) $$\begin{array}{l} \beta_{m}=4\varphi e^{\left(-\left(V_{m}-V_{rest}\right)\right)/20} \end{array}$$

(20) $$\begin{array}{l} \beta_{n}=0.125\varphi e^{\left(-\left(V_{m}-V_{rest}\right)\right)/80} \end{array}$$

(21) $$\begin{array}{l} \beta_{h}=\varphi /\left(e^{\left(3.0-0.1\left(V_{m}-V_{rest}\right)\right)}+1\right) \end{array}$$

**Figure 2:**
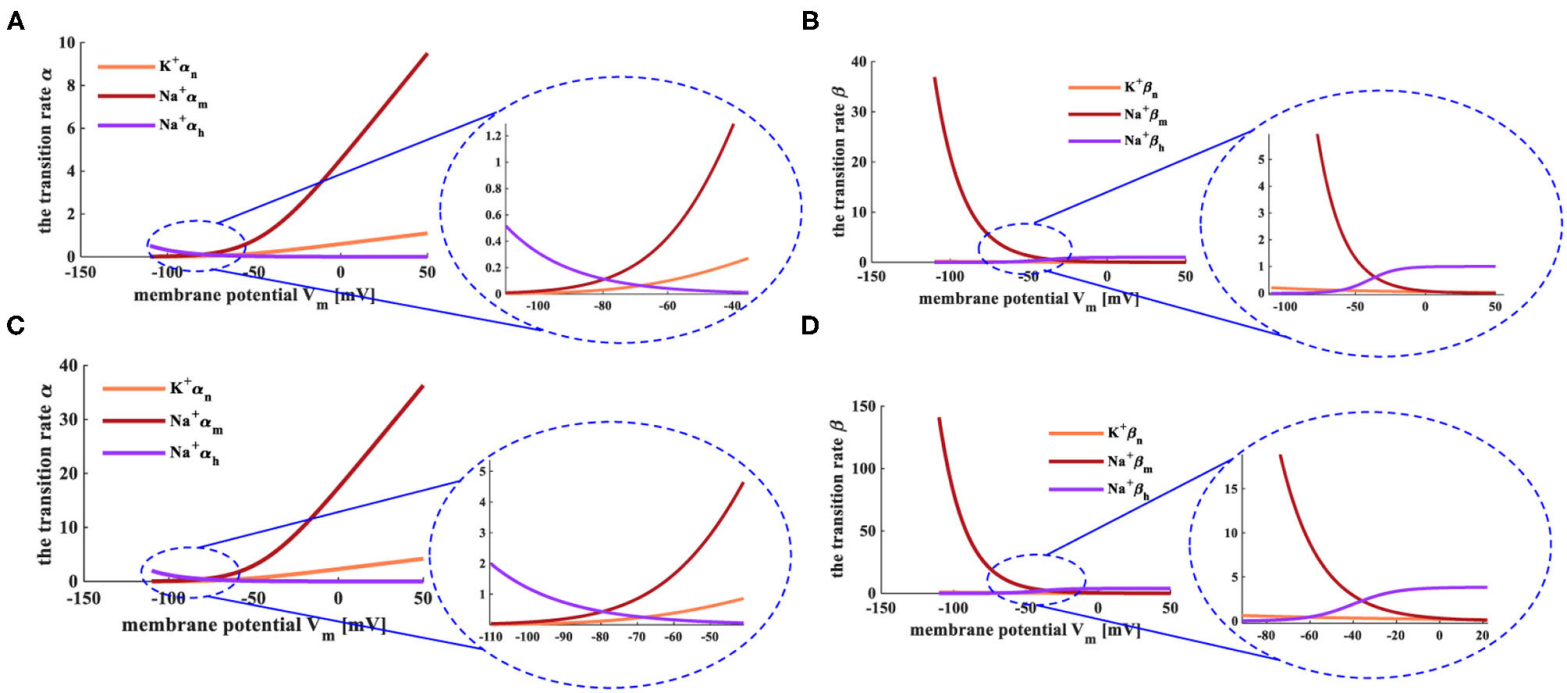
The relationship between transition state and membrane potential. **(A)** The evolution of the transition rate α at the temperature of 6.3°C. **(B)** The variation of the transition rate β at the temperature of 6.3°C. **(C)** The evolution of the transition rate α at the temperature of 18.5°C. **(D)** The change of the transition rate β at the temperature of 18.5°C.

Here, φ=3^(*T*−6.3)/10^. The relationship between the transition state and the membrane potential is shown in Figure 2 (Hodgkin and Huxley, 1952, 1989; Börgers, 2017).

The HH spiking neuron model is strongly dependent on the temperature, and the early experiments were carried out under the temperatures *T* = 6.3°*C* and *T* = 18.5°*C*. When the temperature is 6.3°*C*, the transition rates of the active gates α_*n*_ and α_*m*_ (Figure 2A), the inactive rate β_*h*_ (Figure 2B) increase with the rise of the membrane potential. The inactive transition rate α_*h*_ (Figure 2A), the active transition rates β_*n*_ and β_*m*_ (Figure 2B) decrease with the increase of the membrane potential. When the temperature is increased to 18.5°*C*, the transition rates α and β show the same experimental phenomena (Figures 2C,D) as above. We compare the transition rates at different temperatures, and the difference is performed in the light blue ellipse. When the temperature is 6.3°*C*, α_*n*_ varies from 0 to 10, α_*m*_ alters from 0 to 1, α_*h*_ changes from 0.5 to 0 (the enlarged plot in Figure 2A). When the temperature is 18.5°*C*, α_*n*_ varies from 0 to 36, α_*m*_ adjusts from 0 to 3.5, α_*h*_ changes from 2 to 0 (the enlarged plot in Figure 2C). When the temperature is set to 6.3°*C*, β_*n*_ varies from 37 to 0, β_*m*_ adjusts from 0.2 to 0, β_*h*_ changes from 0 to 1 (the enlarged plot in Figure 2B). When the temperature is increased to 18.5°*C*, β_*n*_ varies from 140 to 0, β_*m*_ adjusts from 0.8 to 0, β_*h*_ changes from 0 to 4 (the enlarged plot in Figure 2D). The higher the temperature, the greater the range of conversion rates, the longer time needed to return to the critical value of the transition rate.

**Figure 3:**
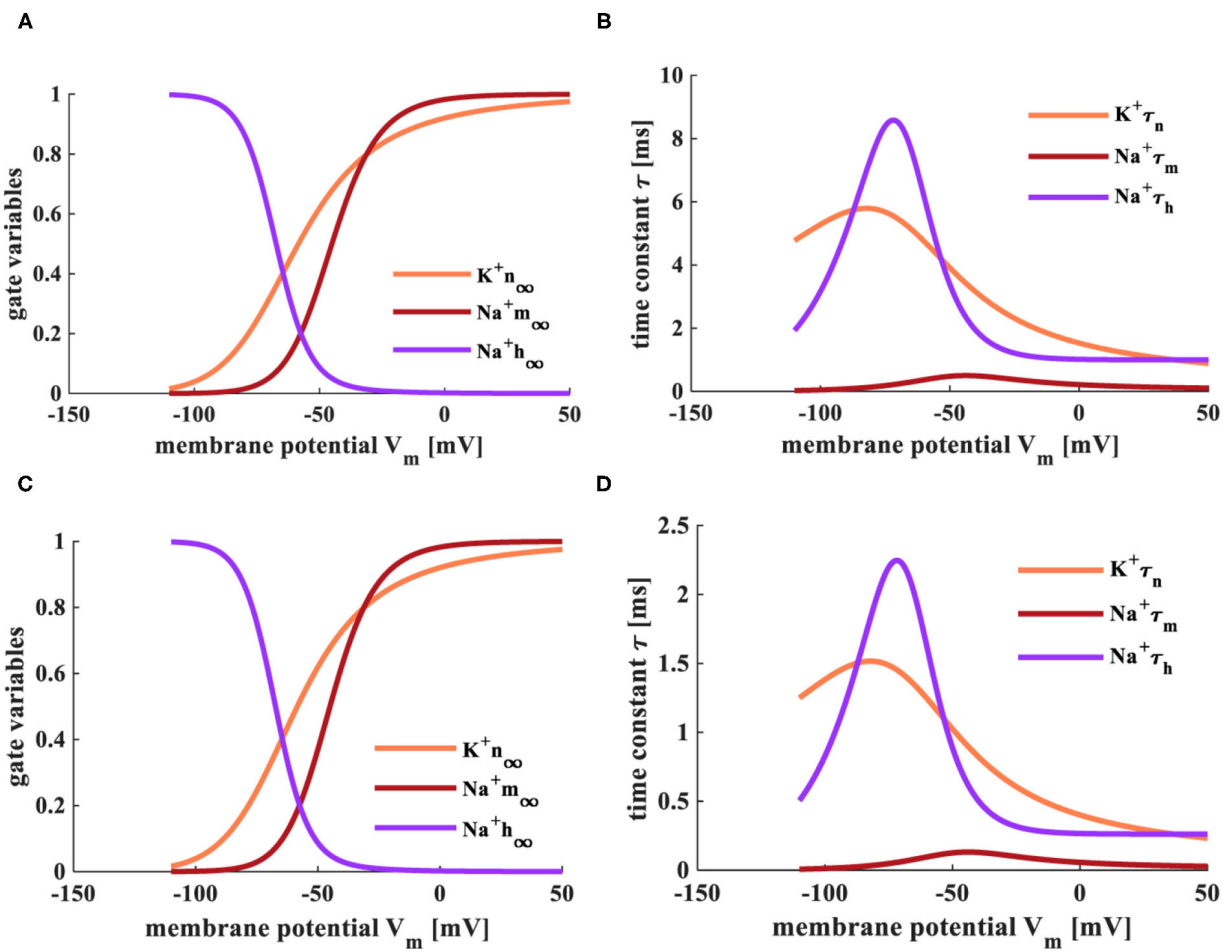
The relationship between gate-controlled variables, the time constant, and the membrane potential. **(A)** The evolution of the gate-controlled variables (m, n, and h) at the temperature of 6.3°*C*. **(B)** The change of the time constant (τ) at the temperature of 6.3°*C*. **(C)** The change of the gate-controlled variables (m, n, and h) at the temperature of 18.5°*C*. **(D)** The evolution of the time constant (τ) at the temperature of 18.5°*C*.

When the temperatures are *T* = 6.3°*C* and *T* = 18.5°*C*, the simulation plots between the steady values of gate variables (*m*_∞_, *n*_∞_, and *h*_∞_) and the membrane potential, the relationship between the time constant (τ_*m*_, τ_*n*_, and τ_*h*_) and the membrane potential, as shown in Figure 3.

The steady-state values (*m*_∞_ and *n*_∞_) of activation gate variables (m and n) change from 0 to 1 with the increase of the membrane potential. The steady-state value (*h*_∞_) of the inactivation gate variable (h) decreases with the increase of the membrane potential (Figures 3A,C). The steady-state values are not affected by the change of temperature. When the temperature is 6.3°C, τ_*n*_ varies from 5.8 to 1, τ_*m*_ adjusts from 0.8 to 0, τ_*h*_ changes from 9 to 1. When the temperature is increased to 18.5°C, τ_*n*_ varies from 1.5 to 0.25, τ_*m*_ adjusts from 0.2 to 0, τ_*h*_ changes from 2.25 to 0.25 (Figures 3B,D). The higher temperature, the smaller the range of τ.

## 3. The Electrical Circuit of the Hodgkin-Huxley Spiking Neuron

**Figure 4:**
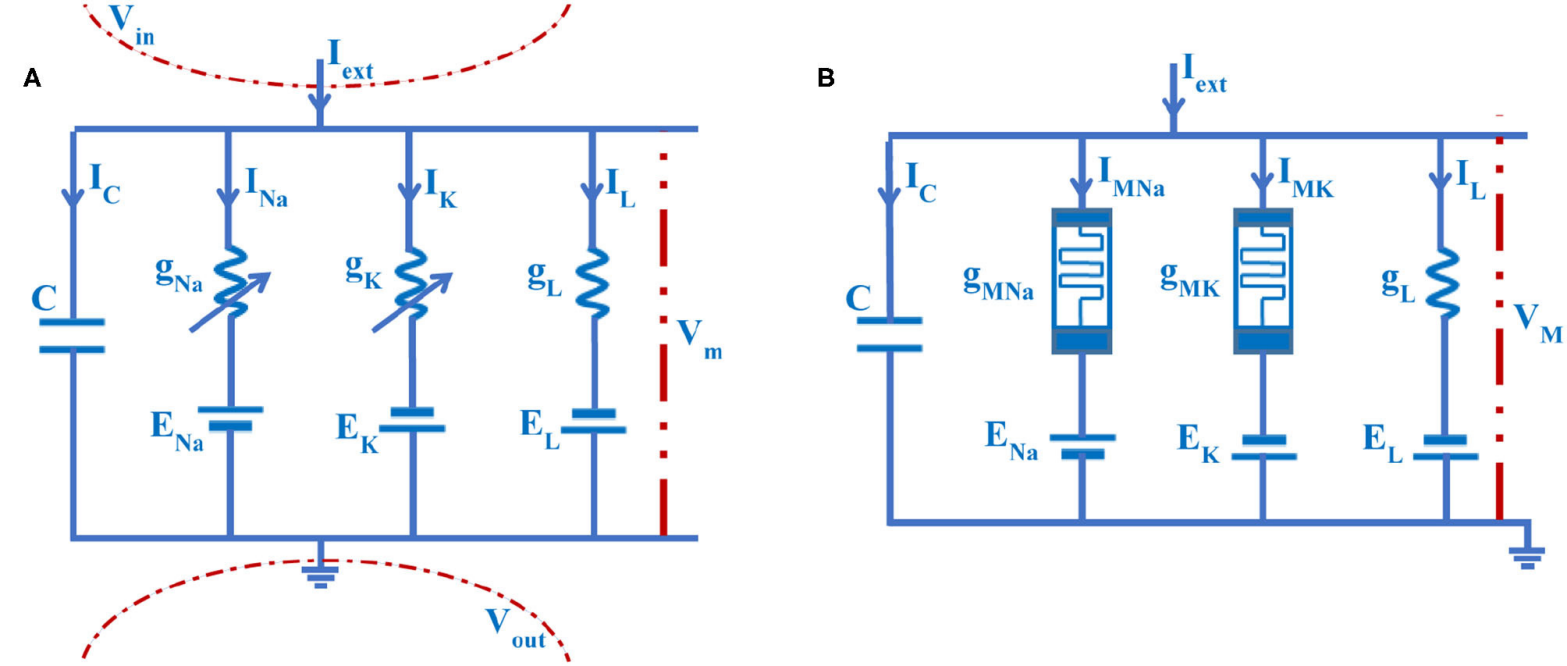
The electrical circuit of the axon cell membrane. **(A)** The HH circuit model. **(B)** The MHH circuit model.

The significant electrical properties of a neuron can be precisely replicated by the HH circuit model, as shown in Figure 4A (Hodgkin and Huxley, 1989).

Here, C is the membrane capacitor. *g*_*Na*_ is the sodium conductance, *g*_*K*_ is the potassium conductance, and *g*_*L*_ is the leaky conductance. *V*_*m*_ is the membrane potential. *I*_*C*_ is the capacitor current, *I*_*Na*_ is the sodium current, *I*_*K*_ is the potassium current, and *I*_*L*_ is the leaky current. *I*_*ext*_ is the external stimulus. *E*_*Na*_, *E*_*K*_, and *E*_*L*_ are ion concentration differences of sodium, potassium, and leakage [namely, the equilibrium potentials (Emili et al., 2003) are calculated by the Nernst equation (Hill, 1992)]. The arrow directions of currents are pointing from inside to outside of the membrane. The value of the extracellular potential is set to zero (*V*_*out*_= 0, namely, the extracellular is grounded) (Hodgkin and Huxley, 1989).

According to Kirchhoff's voltage-current law, the circuit equations are described as:


(22) $$\begin{array}{l} V_{m}=V_{in}-V_{out} \end{array}$$

(23) $$\begin{array}{l} I_{C}=dQ/dt \end{array}$$

(24) $$\begin{array}{l} Q=CV_{m} \end{array}$$

(25) $$\begin{array}{l} I_{m}=I_{Na}+I_{K}+I_{L} \end{array}$$

(26) $$\begin{array}{l} I_{ext}=I_{C}+I_{Na}+I_{K}+I_{L}=I_{C}+I_{m} \end{array}$$

In the giant squid axon experiment, the current through the axon membrane is expressed as the current density J(t, x). It represents the amount of the electric current per square centimeter, and its unit is *mAcm*^−2^. Based on the mathematical analysis of the RC equivalent circuit (Figure 4A), the following voltage-current equations are obtained.


(27) $$\begin{array}{l} C\partial V_{m}\left(t,x\right)/\partial t=-J_{m}\left(t,x\right)+J_{ext}\left(t,x\right)+1/\left(2r_{in}\right)\partial^{2}V_{m}\left(t\right)/\partial x^{2} \end{array}$$

(28) $$\begin{array}{l} J_{m}=J_{Na}+J_{K}+J_{L} \end{array}$$

(29) $$\begin{array}{l} J_{Na}=g_{Na}\left(V_{m}-E_{Na}\right) \end{array}$$

(30) $$\begin{array}{l} J_{K}=g_{K}\left(V_{m}-E_{K}\right) \end{array}$$

(31) $$\begin{array}{l} J_{L}=g_{L}\left(V_{m}-E_{L}\right) \end{array}$$

The left side of (27) is the charging or discharging rate per unit area for the capacitor. *J*_*m*_(*t, x*) is the total current density that flows through the membrane. *J*_*Na*_ is the current density passing through sodium conductance. *J*_*K*_ is the current density of potassium. *V*_*m*_ is the membrane potential. *J*_*ext*_(t, x) is the external stimulus. The last term is the charge rate of longitudinal current along the inside membrane surface. It depends only on the time t rather than the location x, so the quadratic partial differential term equals zero, (27) can be rewritten as:


(32) $$\begin{array}{l} C\partial V_{m}\left(t,x\right)/\partial t=-J_{m}\left(t,x\right)+J_{ext}\left(t,x\right) \end{array}$$

The propagated action potential is performed by (32). The action potential is sensitive to the temperature. The action potential of the cell membrane shows distinct firing behaviors under various temperatures.

**Figure 5:**
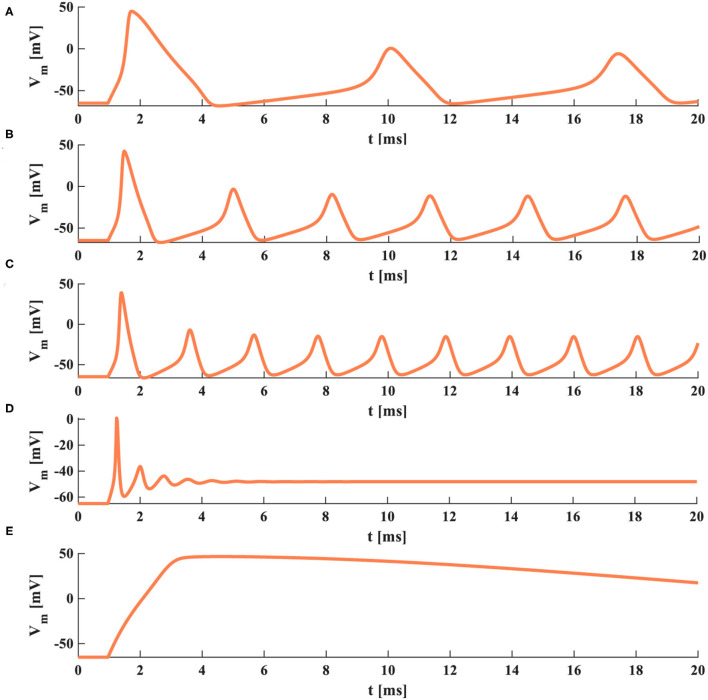
The action potentials under the different temperatures (the external stimulus is 0.08 *mAcm*^−2^). **(A)** The temperature is 6.3°*C*. **(B)** The temperature is 15°*C*. **(C)** The temperature is 20°*C*. **(D)** The temperature is 35°*C*. **(E)** The temperature is −20°*C*.

When the temperature is 6.3°*C*, the HH spiking model generates three action potentials in 20 ms, the duration of a spike is 7.65 ms (Figure 5A). When the temperature becomes 15°*C*, the HH spiking model generates six action potentials in 20 ms, the duration of a spike decreases to 3.35 ms (Figure 5B). When the temperature is increased to 20°*C*, the HH spiking model generates nine action potentials in 20 ms, the duration of a spike reduces to 1.95 ms (Figure 5C). We increase the temperature to 35°*C*, and there is no action potential produced after one action potential is generated (Figure 5D). We decrease the temperature to −20°*C*, and the action potential cannot be obtained (Figure 5E). The temperature affects the time duration of the spike, the generation of action potentials, and the firing frequency of a neuron. It is hard to achieve the action potential when the temperature is too high or low. The increase of temperature has significantly decreased the time duration of the spike and remarkably produced a higher firing frequency.

**Figure 6:**
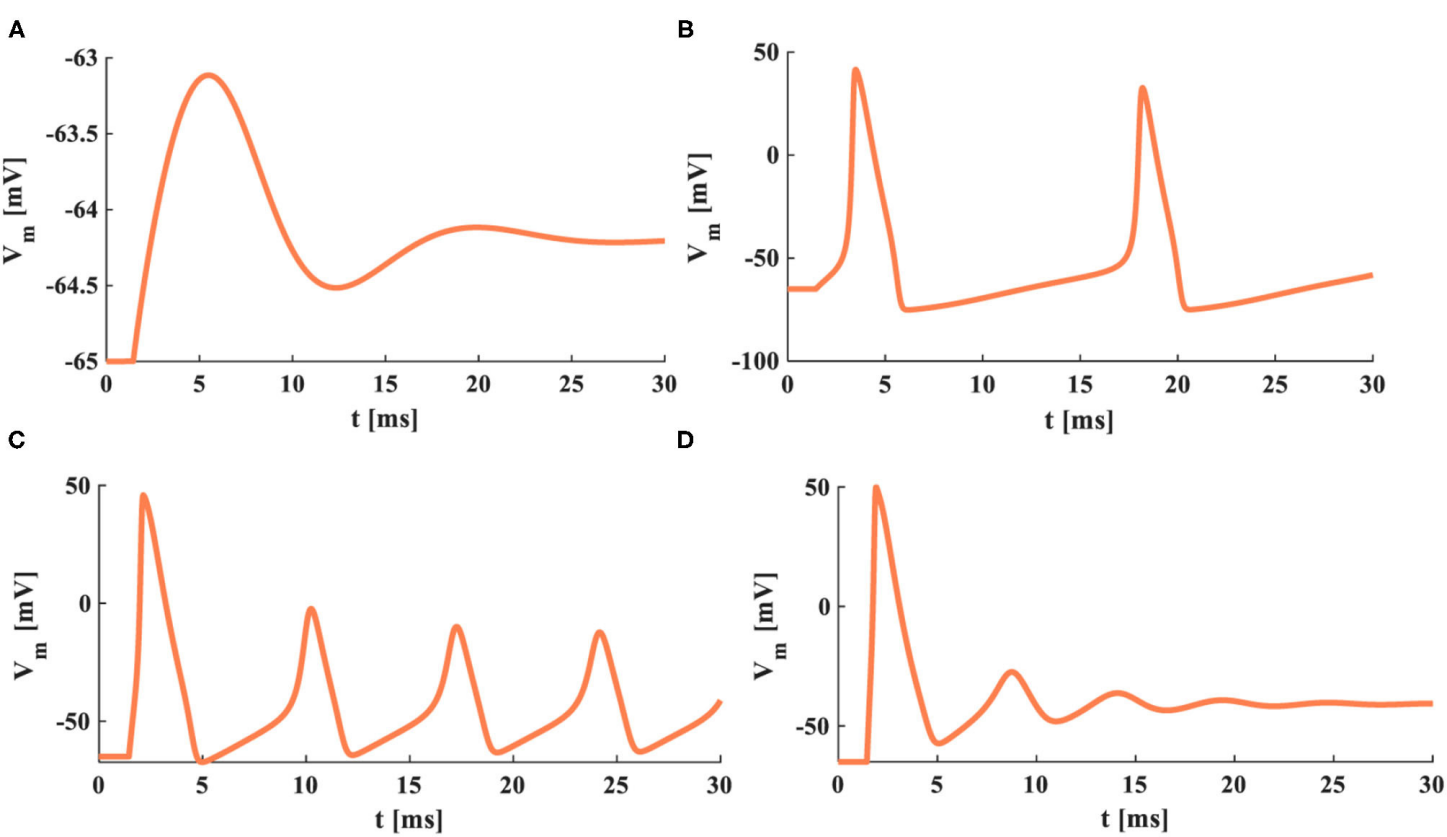
The distinct stimuli are applied to the HH model. **(A)** The external stimulus is 0.001 *mAcm*^−2^. **(B)** The external stimulus is 0.01 *mAcm*^−2^. **(C)** The external stimulus is 0.09 *mAcm*^−2^. **(D)** The external stimulus is 0.2 *mAcm*^−2^.

The external stimuli with various intensities act on the HH spiking model, which performs different action potentials. When the current density is 0.001*mAcm*^−2^, the HH spiking model cannot produce the action potential (Figure 6A). When the current densities are increased to 0.01 and 0.09*mAcm*^−2^, the action potentials are obtained (Figures 6B,C). However, when the current density becomes 0.2*mAcm*^−2^, the HH spiking model generates one action potential. After that, it cannot produce the action potentials (Figure 6D). The external stimulus is related to the generation of the action potential. The larger the external stimulus, the higher the firing frequency. If the external stimulus is too larger or small, the HH spiking model cannot reproduce the action potential.

**Figure 7:**
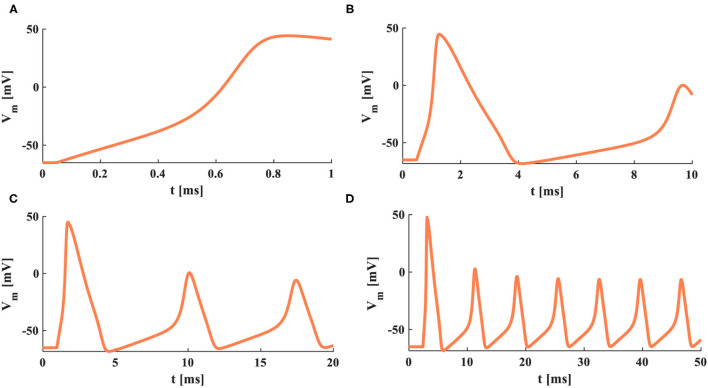
The firing behaviors under the various action time of the external stimulus. **(A)** The action time is 1 ms. **(B)** The action time is 10 ms. **(C)** The action time is 20 ms. **(D)** The action time is 50 ms.

When the action time of the external stimulus is 1 ms, there is not enough time to show the complete firing process (Figure 7A). Therefore, the action time is increased to 10 ms, and the action potential is generated (Figure 7B). When the action time becomes 20 or 50 ms, the HH spiking model produces more action potentials (Figures 7C,D). Thus, the action time of the external stimulus has a strong influence on the generation of the action potential. The longer the action time, the more action potentials generated. But when the action time is too long or short, the HH spiking model cannot perform the firing process.

## 4. The Memristive Hodgkin-Huxley (MHH) Spiking Neuron Model

In the HH circuit model, the potassium conductance and the sodium conductance are voltage-gated channels, which can be described by time and membrane potential. The flux-controlled memristor with the nonvolatile property is the function of time and voltage, which can be used in a nonlinear circuit system (Petras, 2010; Corinto and Forti, 2017; Corinto et al., 2018). Based on the HH spiking model, we replace the sodium and potassium conductances with the flux-controlled memristors (Wang et al., 2012), and the memristive Hodgkin-Huxley spiking neuron model is constructed (Figure 4B).

Some of the mathematical expressions in the HH spiking model need to be modified. *g*_*Na*_ and *g*_*K*_ in (4) and (5) are replaced by the memristance and rewritten as:


(33) $$\begin{array}{l} g_{MK}=1/M_{K}n^{4} \end{array}$$

(34) $$\begin{array}{l} g_{MNa}=1/M_{Na}m^{3}h \end{array}$$

The conductance values of the sodium and potassium ion channels become the function of time, and the membrane potential will change with the evolution of the memristance.

The flux-controlled memristor is described as (Wang et al., 2012):


(35) $$M(\phi (t))=\{\begin{array}{ll} 20000 & \phi (t)<-0.75\\ \sqrt{-3.98\times 10^{8}\phi (t)+10^{8}} & \phi (t)\geq -0.75\text{ and}\\ \; & \phi (t)<0.25\\ 100 & \phi (t))\geq 0.25 \end{array}$$

**Figure 8:**
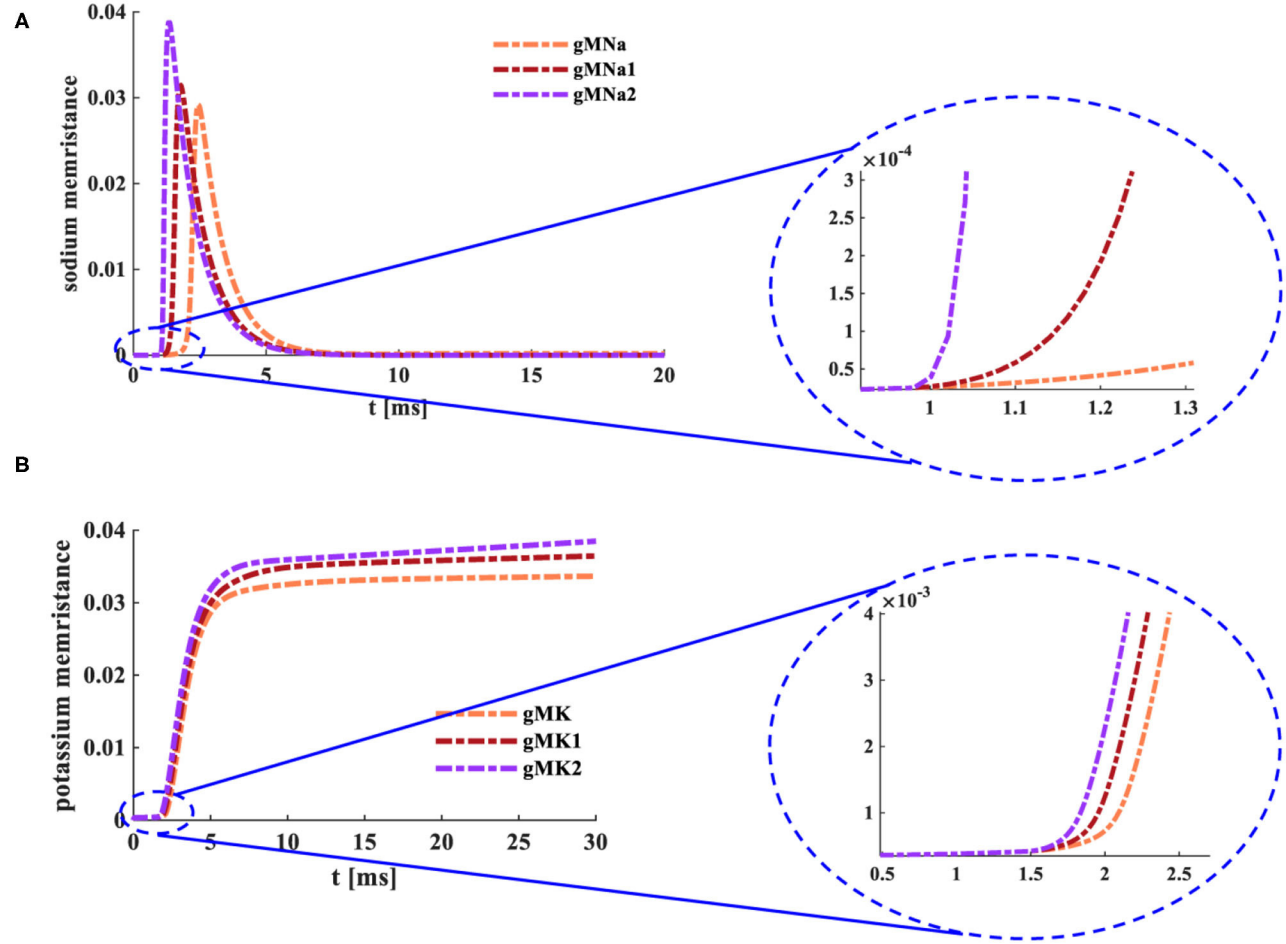
Changes of the memristance under the distinct input currents. **(A)** The variations of the sodium memristance. **(B)** The variations of the potassium memristance.

Where *M*_*K*_=*M*_*Na*_=M is the function of time. The potassium memristance (*g*_*MK*_) and the sodium memristance (*g*_*MNa*_) are functions involved with time and membrane potential. When the various external stimuli act on the MHH spiking neuron model, changes in *g*_*MK*_ and *g*_*MNa*_ are performed in Figure 8.

The initial values of memductances and reductance *g*_*MK*_= 0.5×10^−4^ *Ohm*^−1^*cm*^−2^, *g*_*MNa*_= 0.5×10^−4^ *Ohm*^−1^*cm*^−2^, and *g*_*L*_= 0.3×10^−3^ *Ohm*^−1^*cm*^−2^ [0.5×10^−4^ is the reciprocal of the maximum value (20,000 *Ohmcm*^−2^) of a memristor]. The temperature is 6.3°C, C is 1 μF *m*^−2^. *E*_*Na*_ is 50 mV, *E*_*K*_ is −70 mV, and *E*_*L*_ is −50 mV.

When the external stimulus [0.008 mA *cm*^−2^ (*g*_*MNa*_)] is applied to the MHH spiking model, the sodium memductance (the coral color curve) does not change in the time range from 0 to 1.025 ms (the enlarged plot in Figure 8A). Then, the sodium memductance increases to 0.029 *Ohm*^−1^*cm*^−2^ and then decreases to zero. When the MHH spiking model receives the external stimulus [0.08 *mAcm*^−2^ (*g*_*MNa*1_)], the sodium memductance (the dark red curve) remains the same in the time range from 0ms to 1.38ms (the enlarged plot in Figure 8A). And the maximum value of the sodium memductance is 0.031 *Ohm*^−1^*cm*^−2^. Likewise, when the external stimulus [0.8 *mAcm*^−2^ (*g*_*MNa*2_)] acts on the MHH spiking model, the sodium memductance (the purple curve) does not change in the time range from 0 to 0.97 ms (the enlarged plot in Figure 8A). And the maximum value of the sodium memductance is 0.038 *Ohm*^−1^*cm*^−2^.

When the external stimulus [0.04 *mAcm*^−2^ (*g*_*MK*_)] is injected into the MHH spiking model, the potassium memductance (the coral color curve) does not change from 0 to 1.5 ms (the enlarged plot in Figure 8B). Then, the potassium memductance increases and attains 0.0324 *Ohm*^−1^*cm*^−2^. Likewise, the MHH spiking model receives the external stimuli [(0.08 *mAcm*^−2^ (*g*_*MK*1_) and 0.16 *mAcm*^−2^ (*g*_*MK*2_)], the potassium memductance (the dark red curve reaches 0.0348 *Ohm*^−1^*cm*^−2^ and the purple curve attains 0.0359 *Ohm*^−1^*cm*^−2^ (the enlarged plot in Figure 8B) are stable at constant values (Figure 8B).

The sodium memductance and the potassium memductance are associated with the external stimulus. The stronger the external input, the faster the memductance changes, the larger the memductance value. The change curves of sodium and potassium memductance are similar to the theoretical curves (refer to Hodgkin and Huxley, 1989). Therefore, the memristors can mimic the sodium ion channel and the potassium ion channel.

**Figure 9:**
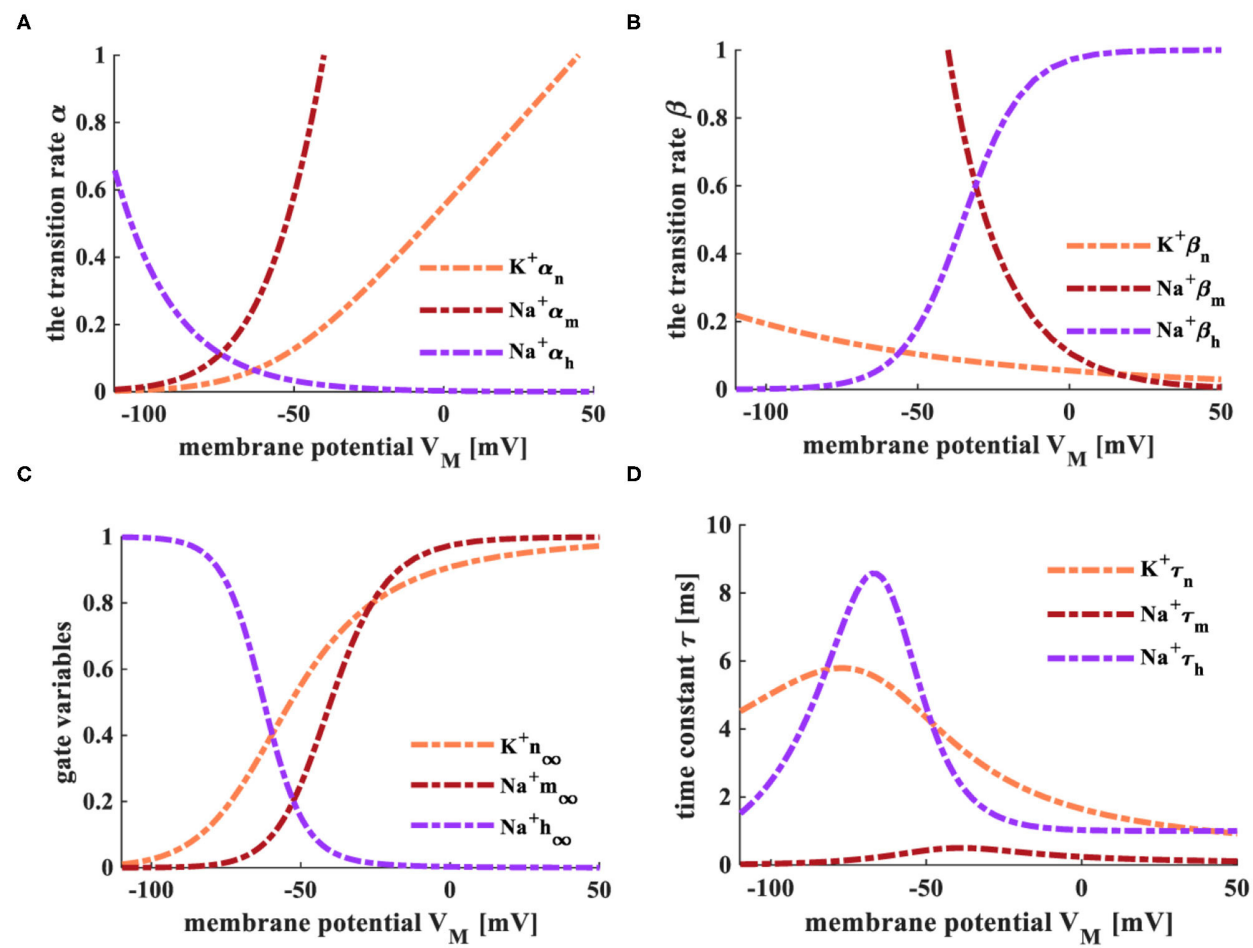
The transition rate, gate variables, and time constant of the MHH spiking model. **(A)** The variation process of the transition α. **(B)** The variation process of the transition rate β. **(C)** The variation process of gate variables. **(D)** The variation of the time constant τ.

The temperature is selected as 6.3°C, and the external current is 0.08 *mAcm*^−2^. The transition rate parameters (α and β), gate variables (*m*_∞_, *n*_∞_, and *h*_∞_), and the time constant (τ) in the MHH spiking model are shown in Figure 9.

The transition rates of the active gates (α_*n*_ and α_*m*_, Figure 9A), the inactive transiton rate (β_*h*_, Figure 9B) enhance with the increase of the membrane potential. The inactive transition rate (α_*h*_, Figure 9A), the active transition rates (β_*n*_ and β_*m*_, Figure 9B) decrease with the rise in the membrane potential. The steady-state values (*m*_∞_ and *n*_∞_) of activation gate variables (m and n) change from 0 to 1 with the increase of the membrane potential. The steady-state value (*h*_∞_) of the inactivation gate variable (h) decreases with the increase of the membrane potential (Figure 9C). The time constant τ_*n*_ changes from 4.52 to 0, τ_*m*_ adjusts from 0.5 to 0, and τ_*h*_ varies from 8.57 to 0 (Figure 9D). The changing processes of the transition rate, gate variables, and the time constant in the MHH spiking model have high similarities with those of the HH spiking model in Figures 2, 3. Therefore, the memristors can be utilized as the sodium ion channel and the potassium ion channel.

When the current density *J*_*m*_ in (28) is replaced by *J*_*M*_, conductances *g*_*Na*_ and *g*_*K*_ in (29) and (30) are replaced by *g*_*MNa*_ and *g*_*MK*_, and the current equations are rewritten as:


(36) $$\begin{array}{l} J_{M}=J_{MNa}+J_{MK}+J_{L} \end{array}$$

(37) $$\begin{array}{l} J_{MNa}=g_{MNa}\left(V-E_{Na}\right) \end{array}$$

(38) $$\begin{array}{l} J_{MK}=g_{MK}\left(V-E_{K}\right) \end{array}$$

The membrane potential *V*_*m*_ in (32) is replaced by *V*_*M*_, and the membrane potential of the MHH spiking neuron model is described as:


(39) $$\begin{array}{l} C\partial V_{M}\left(t,x\right)/\partial t=-J_{M}\left(t,x\right)+J_{ext}\left(t,x\right) \end{array}$$

The electrical equivalent circuit of the HH spiking model is based on the voltage-clamp experimental method. When the voltage-clamp values are distinct, the variables perform various variations in the HH and MHH spiking models. Here, the temperature T = 6.3°C. The clamp voltage is denoted by *V*_*clamp*_, and its value is selected as +20 or +80 mV. The resting potential *V*_*rest*_ = −65 mV. The membrane potential *V*_*m*_=*V*_*clamp*_ +*V*_*rest*_.

**Figure 10:**
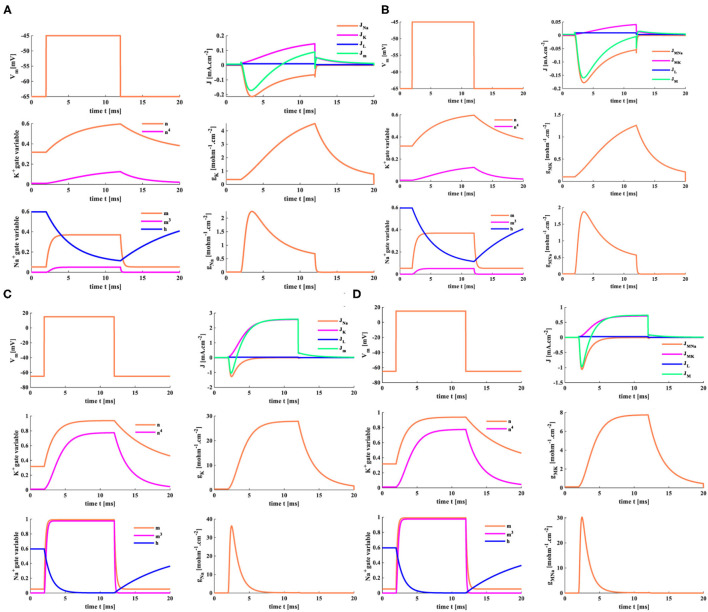
The distinct clamp voltages are applied to the HH spiking model and the MHH spiking model. **(A)** The HH model with *V*_*clamp*_ = 20 mV. **(B)** The MHH model with *V*_*clamp*_ = 20 mV. **(C)** The HH model with *V*_*clamp*_ = 80 mV. **(D)** The MHH model with *V*_*clamp*_ = 80 mV.

When the clamp-voltage value is 20 mV, the membrane potential becomes −45 mV. Changes of *Na*^+^ and *K*^+^ gate variables in the MHH spiking model (the plots on the left in Figure 10B) are the same as those in the HH spiking model (the plots on the left in Figure 10A). The HH spiking model generates the reverse curves of *J*_*Na*_ and *J*_*m*_, and their maxima are −0.17 and −0.21 *mAcm*^−2^. The maximum of the forward curve *J*_*K*_ is 0.14 *mAcm*^−2^, and the forward curve *J*_*L*_ reaches 0.009 *mAcm*^−2^. The peak values of *g*_*K*_ and *g*_*Na*_ are 4.54 and 2.25 *mOhm*^−1^*cm*^−2^ (the plots on the right in Figure 10A). The MHH spiking model produces the reverse curves of *J*_*MNa*_ and *J*_*M*_, and their maxima are −0.18 and −0.16 *mAcm*^−2^. The forward curves of *J*_*MK*_ and *J*_*L*_ attain their maxima 0.04 and 0.009 *mAcm*^−2^. The maxima of *g*_*MK*_ and *g*_*MNa*_ are 1.26 and 1.88 *mOhm*^−1^*cm*^−2^ (the plots on the right in Figure 10B).

The variable values of the HH spiking model are more significant than those of the MHH spiking model (because the memristance is large, its initial value is 10,000 *Ohmcm*^−2^). When the clamp-voltage value is 20 mV, both spiking models cannot generate the action potential.

A transient increase of sodium ions in the cell leads to the depolarization of the action potential. The waveforms of the two models change in the same way when the clamp voltage is 80 mV (the membrane potential is 15 mV). We take the MHH model as an example and make a vertical comparison (Figures 10B,D). With the increase of clamp voltage, the current densities of sodium and potassium increase significantly. The value of gate variable n changes from 0.5 to 1, and the value of gate variable m varies from 0.4 to 1. The potassium memductance changes from 1.26 to 8 *mOhm*^−1^*cm*^−2^, and the sodium memductance changes from 1.88 to 30 *mOhm*^−1^*cm*^−2^.

When the clamp-voltage value is 80 mV, the HH and MHH spiking models can produce the action potential. The gate variables n and m change with the identical waveforms. The current densities, the potassium conductance, and the sodium conductance are different. The maxima of *J*_*MNa*_, *J*_*MK*_, *J*_*L*_, and *J*_*M*_ are −1.059, 0.74, 0.0297, and −0.97 *mAcm*^−2^ (the right-upper plot in Figure 10D), which are larger than those of the HH spiking model (Figure 10C). The variation ranges of potassium conductance and sodium conductance for the MHH spiking model are [0 8], [0 30] less than those [0 29], [0 37] in the HH spiking model. The higher the voltage-clamp value, the larger the variable values, the smaller the conductance variation range.

## 5. The Comparison Between Two Models Under the Different Stimuli

### 5.1. The Individual Current Pulse Stimulus

The forward stimulus *J*_*ext*_ = 0.1*mAcm*^−2^ (the pulse width is 0.1 ms) is applied to the HH spiking model and the MHH spiking model, the temperature is selected as 18.5°C, and the response time of the model is 5 ms. The initial value of the membrane potential is the resting potential, *V*_*rest*_ = −65 mV.

Here, *J*_*ext*_ is the external stimulus, *J*_*Na*_ (*J*_*MNa*_) is the sodium current (the coral color curve), *J*_*K*_ (*J*_*MK*_) is the potassium current (the blue curve), *J*_*L*_ (*J*_*ML*_) is the leaky current (the green curve), and *J*_*m*_ (*J*_*M*_) is the total current (the purple curve) flowing through the cell membrane in the HH (MHH) spiking model. V (*V*_*M*_) is the action potential generated by the HH (MHH) spiking model. *g*_*Na*_ (*g*_*MNa*_) is the sodium conductance (the sodium memductance), and *g*_*K*_ (*g*_*MK*_) is the potassium conductance (the potassium memductance) in the HH (MHH) spiking model.

**Figure 11:**
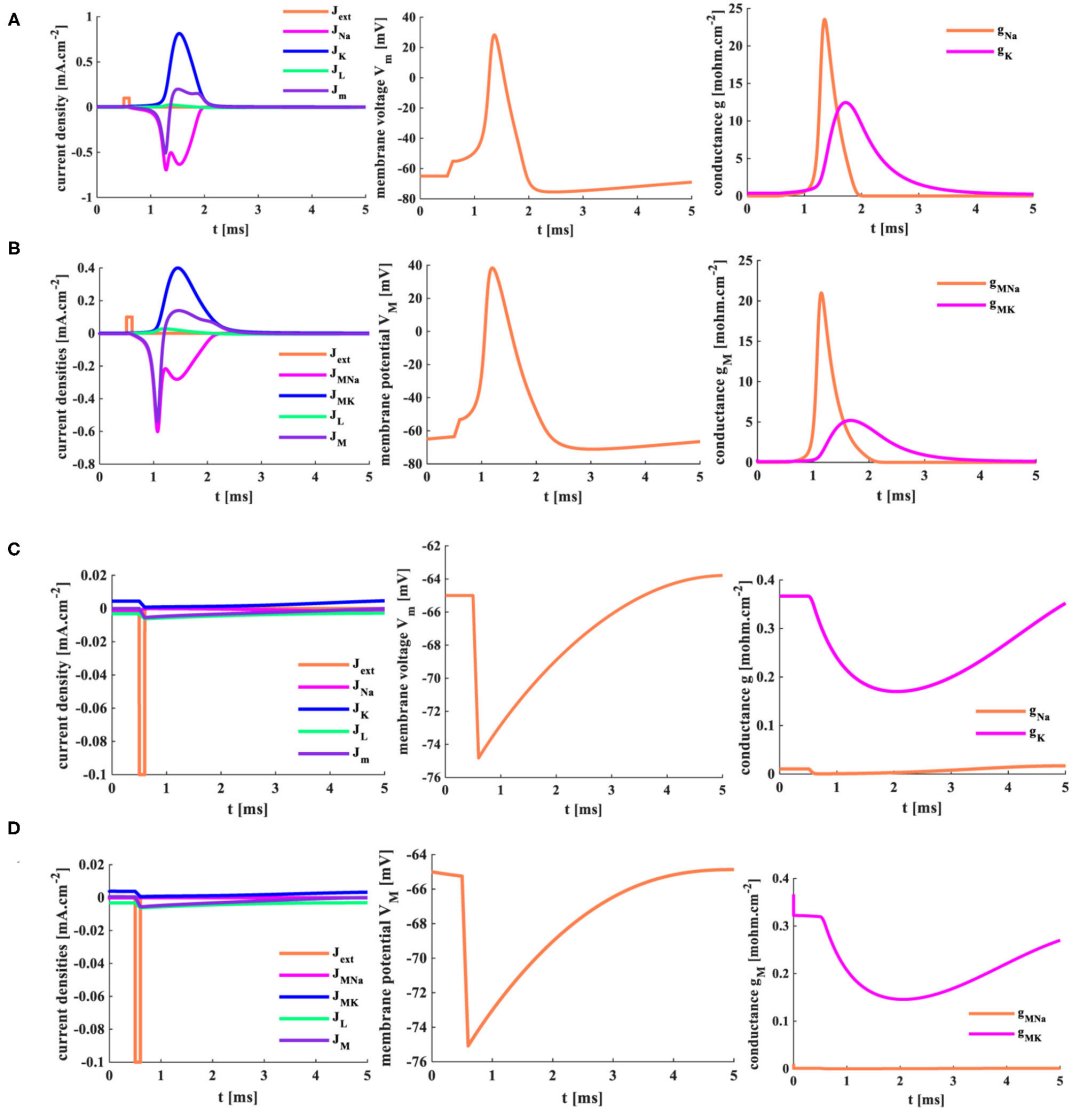
The single pulse and the reverse pules are applied to two models. **(A)** The single current pulse is injected into the HH spiking model. **(B)** The single current pulse is injected into the MHH spiking model. **(C)** The reverse current pulse is injected into the HH spiking model. **(D)** The reverse current pulse is injected into the MHH spiking model.

The HH and MHH spiking models receive the external stimuli and produce the corresponding current densities of the ion channels. The sodium current is negative because the sodium ions move from the outside to the inside of the cell. In contrast, the potassium current is positive because the potassium ions flow from intracellular to extracellular. The potassium and total current densities (the peak values: *J*_*K*_ = 0.82 *mAcm*^−2^, *J*_*m*_= −0.51 *mAcm*^−2^) generated by the HH spiking model are larger than those (the peak values: *J*_*MK*_ = 0.4 *mAcm*^−2^, *J*_*M*_ = −0.53 *mAcm*^−2^) in the MHH spiking model. The sodium and leaky current densities (the peak values: *J*_*Na*_= −0.7 *mAcm*^−2^, *J*_*L*_= 0.024 *mAcm*^−2^) generated by the HH spiking model are smaller than those (the peak values: *J*_*MNa*_= −0.6 *mAcm*^−2^, *J*_*L*_= 0.026 *mAcm*^−2^) in the MHH spiking model. The sodium current of the MHH model has a smooth perturbation at around t = 1.072 s, and the sodium current of the HH model has an obvious perturbation at around t = 1.279 s. The perturbation is caused by the rapid variation of potassium conductance (potassium memductance). The curves formed by the MHH model (the left side plot in Figure 11B) are smoother than those in the HH model (the left side plot in Figure 11A) because the memristor has a unique time-varying property.

The HH spiking model and the MHH spiking model can perform the action potential. The membrane potential peak value (*V*_*M*_ = 38.33 mV at 1.188 ms) of the MHH model (the middle plot in Figure 11B) is stronger than that (*V*_*m*_ = 28.31 mV at 1.366 ms) of the HH model (the central plot in Figure 11A). Meanwhile, the MHH spiking model takes a short time to produce the action potential. After generating the action potential, both models return to the equilibrium state (the resting state, *V*_*rest*_ = −65 mV).

The HH spiking model takes 1.354 ms to reach the maximum value of *g*_*Na*_ (23.53 *mOhm*^−1^*cm*^−2^) and needs 1.715 ms to get the peak value of *g*_*K*_ (12.45 *mOhm*^−1^*cm*^−2^; the right side plot in Figure 11A). Therefore, the MHH spiking model takes 1.134 ms to attain the maximum value of *g*_*MNa*_ (20.81 *mOhm*^−1^*cm*^−2^) and needs 1.673 ms to reach the peak value of *g*_*MK*_ (5.196 *mOhm*^−1^*cm*^−2^) (the right side plot in Figure 11B). The rise in sodium conductance (sodium memductance) is faster than potassium conductance (potassium memductance). The MHH spiking model utilizes less time than the HH model to activate the change of the memductance; however, the obtained memductane is small. Because the variation in the memductance is slight in a short time (5 ms), it maintains a large memristance.

### 5.2. The Reverse Single Current Pulse Stimulus

The reverse stimulus (*J*_*ext*_ = −0.1 *mAcm*^−2^, the pulse width is 0.1 ms) acts on the HH spiking model and the MHH spiking model, the temperature is 18.5°C, and the response time of the model is 5 ms.

There are not enough ions to move from intracellular (extracellular) to extracellular (intracellular); therefore, the sodium current and the potassium current cannot be produced (the left-side plots in Figures 11C,D). The significant variation of the conductance causes the generation of potassium and sodium currents. The sodium conductance (sodium memductance) is close to zero (the right-side plots in Figures 11C,D). The potassium conductance (potassium memductance) decreases from 0.37 *mOhm*^−1^*cm*^−2^ (0.36 *mOhm*^−1^*cm*^−2^) to 0.17 *mOhm*^−1^*cm*^−2^ (0.14 *mOhm*^−1^*cm*^−2^) and then increases to 0.35 *mOhm*^−1^*cm*^−2^ (0.26 *mOhm*^−1^*cm*^−2^). The HH and MHH spiking models are unable to generate the action potential, and the membrane potentials become hyperpolarization before returning to their resting states (the middle plots in Figures 11C,D.

### 5.3. The Three External Stimuli With Different Intensity

The external stimuli *J*_*ext*1_ = 0.5 *mAcm*^−2^, *J*_*ext*2_ =1 *mAcm*^−2^, and *J*_*ext*3_ =2 *mAcm*^−2^ are injected into the HH spiking model and the MHH spiking model, the temperature is 18.5°*C*, the response time is 5 ms.

**Figure 12:**
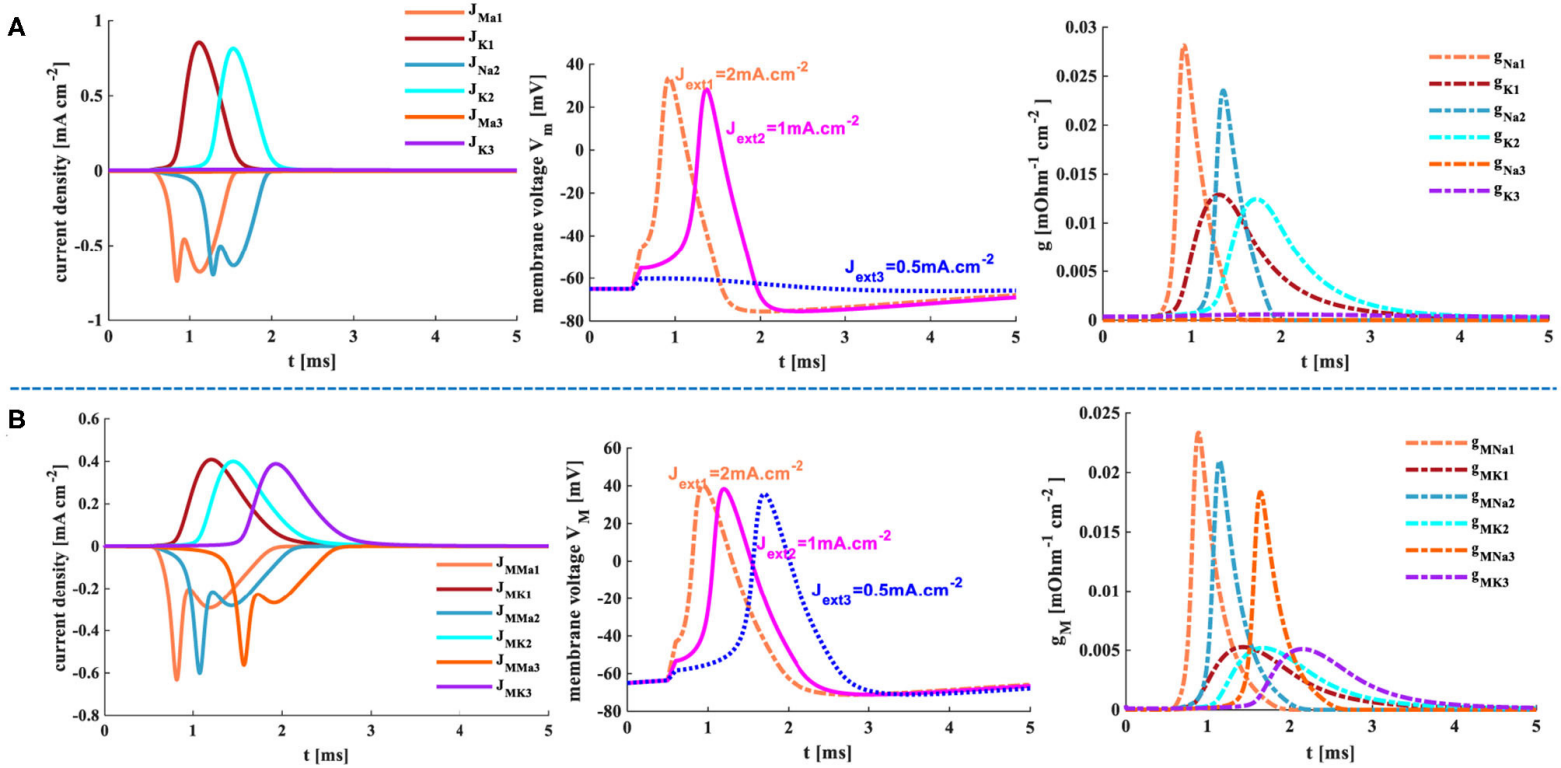
The three different current pulses are applied to the HH and MHH spiking models. **(A)** The HH spiking model. **(B)** The MHH spiking model.

When the small external stimulus (*J*_*ext*1_ = 0.5 *mA*.*cm*^−2^) is applied to the HH spiking model, the action potential cannot be produced. The membrane potential has a slight rise (*V*_*m*_ = −60 mv) and then returns to the resting potential (−65 mv) at 3 ms (the second plot in Figure 12A). The current density is zero (the first plot in Figure 12A). There is only a slight change in the conductance, which can be ignored (the third plot in Figure 12A). However, when the MHH spiking receives the stimulus *J*_*ext*1_ = 0.5 *mA*.*cm*^−2^, the action potential is obtained (the second plot in Figure 12B). The changes in current densities and the memductance are noticeable. When the external stimuli increase to *J*_*ext*2_ = 1 *mA*.*cm*^−2^ and *J*_*ext*1_ = 2 *mA*.*cm*^−2^, the values in current density, membrane potential, and conductances strengthen gradually (Figure 12).

The larger the external stimulus, the faster the action potential is produced, the higher the peak value is generated, the more significant change in conductances, and the greater the current density. The smaller the external stimulus, the longer time it takes to produce the action potential. The peak value of membrane potential in the MHH model (the middle plot in Figure 12B) is greater than that of the HH model (the middle plot in Figure 12A). The maximum values of current densities and conductances in the MHH spiking model (the first and third plots in Figure 12B) are lower than those in the HH spiking model (the first and third plots in Figure 12A).

### 5.4. A Series of Pulse Stimuli

**Figure 13:**
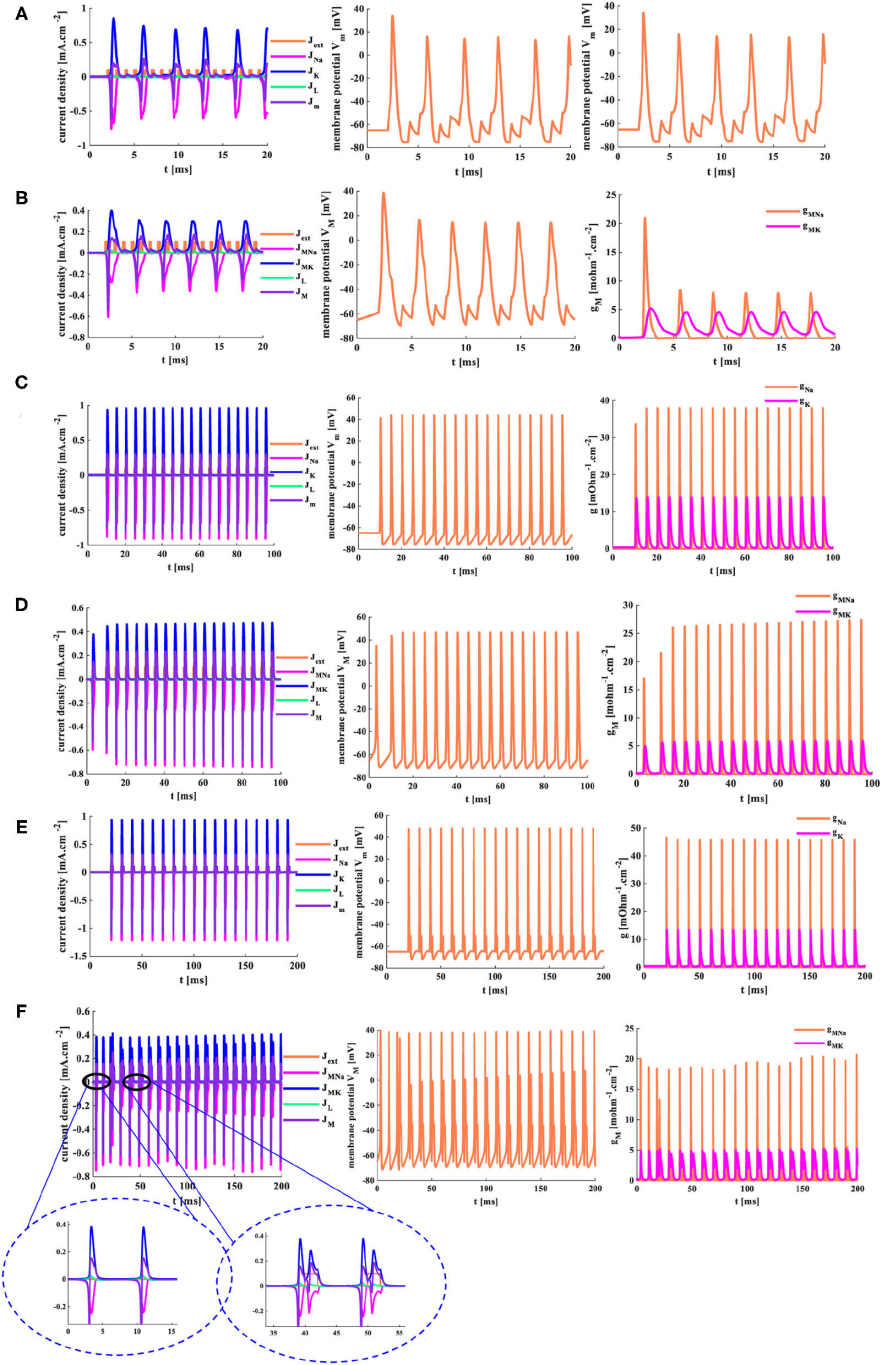
The distinct action time of the external stimulus is set for the two models. **(A)** The HH spiking model with 20 ms action time. **(B)** The MHH spiking model with 20 ms action time. **(C)** The HH spiking model with 100 ms action time. **(D)** The MHH spiking model with 100 ms action time. **(E)** The HH spiking model with 200 ms action time. **(F)** The MHH spiking model with 200 ms action time.

When a series of pulses (*J*_*ext*_(n)= 1*mAcm*^−2^, *n* = 1,2,......,18, the temperature is 18.5°*C*.) act on the HH and MHH spiking models, the action potentials are achieved. However, not every single pulse can cause the generation of the action potential (the first plots in Figures 13A,B). Only when the action potential generated by the previous pulse has enough time to return to its resting state, another action potential will be generated. The MHH spiking model [six action potentials (the second plot in Figure 13B)] generates more action potentials than the HH spiking model (five action potentials (the second plot in Figure 13A)). Meanwhile, the action potential performs two oscillation behaviors in the MHH spiking model (inside the blue ellipse in Figure 13B), and the action potential shows three oscillation behaviors in the HH spiking model (inside the blue ellipse in Figure 13A). The memductances in the MHH model (the third plot in Figure 13B) are smaller than those in the HH model (the third plot in Figure 13A), which causes the current density produced by the MHH model (the first plot in Figure 13B) to be lower than the HH model (the first plot in Figure 13A).

The action time of the external stimulus is extended to 100 ms, and two models can produce more action potentials than Figures 13A,B. The MHH spiking model generates more action potentials (the middle plot in Figure 13C) than the HH spiking model (the middle plot in Figure 13D).

The action time is increased to 200 ms, the doublet currents (Shigaki et al., 2020) are generated in the MHH spiking model, one is large, the other is small (the enlarged plot inside the left ellipse in Figure 13F). Meanwhile, the action potential is produced before the current pulse comes in the MHH model because the memristor has an initial charge even though it is very small (the enlarged plot inside the right ellipse in Figure 13F). The current intensity, the voltage peak value, and conductances in the HH spiking model (Figure 13E) are larger than the simulation results in Figure 13F.

With the increasing of time length, the conductance (or memductance) and the current density of sodium and potassium increase dramatically. The more time we give, the more action potentials are generated, the larger the peak values of current densities, conductances (or memductances), and action potentials. However, the action time length should not be too long; otherwise, the function of neurons cannot be replicated effectively (Chen et al., 2019).

### 5.5. The Sinusoidal Current Stimulus

The sinusoidal stimulus [*J*_*ext*_ = *J*_*extm*_× sin(2 t/*T*_*in*_), *J*_*extm*_=0.01 *mA*.*cm*^−2^] is a positive-negative periodic signal with a single-frequency component. *T*_*in*_ is the time period of input signals, and the temperature is 18.5°*C*.

**Figure 14:**
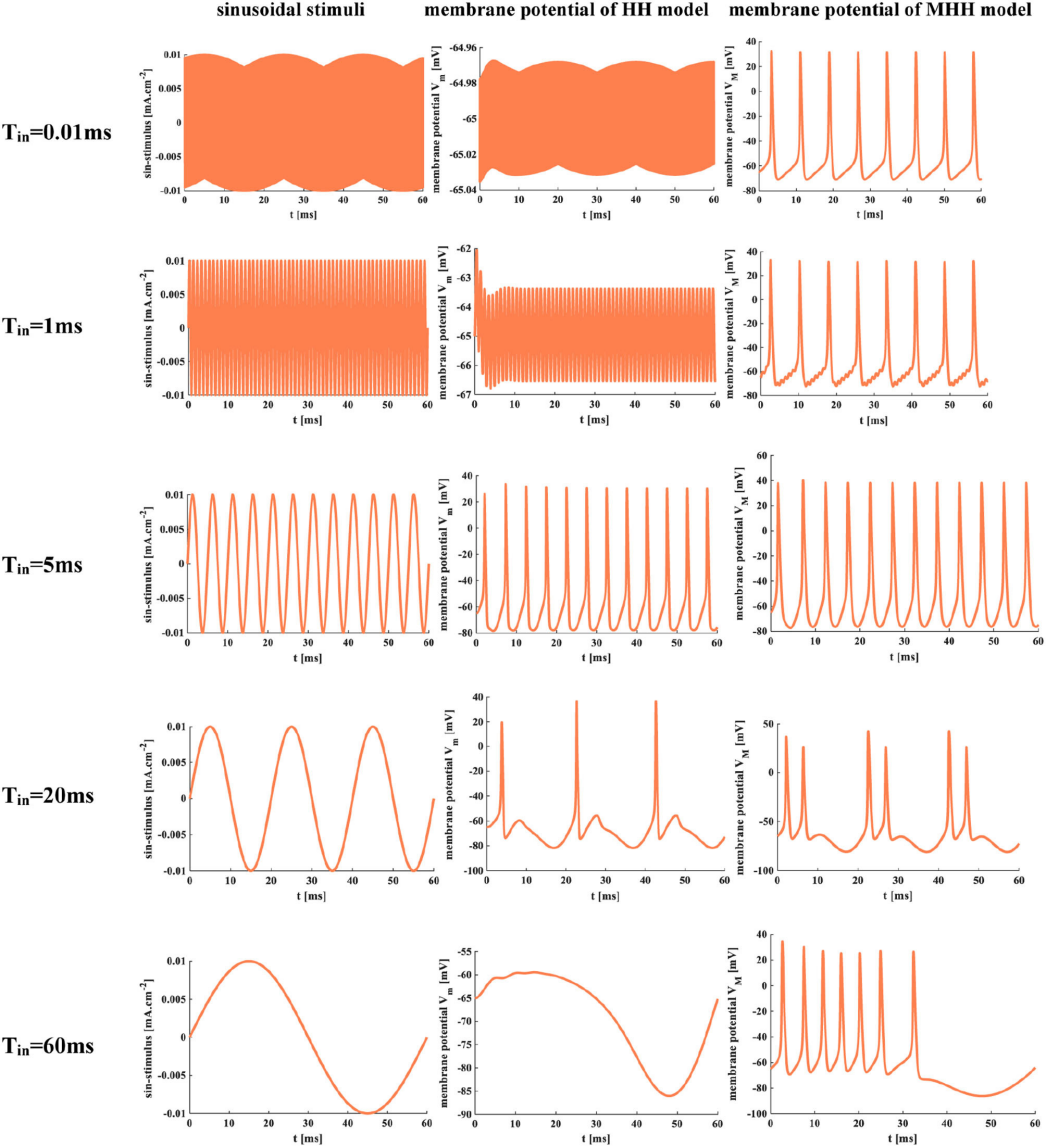
The time periods of the sinusoidal signal. *T*_*in*_ = 0.01 ms, *T*_*in*_ = 1 ms, *T*_*in*_ = 5 ms, *T*_*in*_ = 20 ms, *T*_*in*_ = 60 ms.

When *T*_*in*_ = 0.01 ms and *T*_*in*_ = 1 ms, the sinusoidal stimuli are applied to the HH spiking model. The action potential cannot be obtained because there is not enough time for the neuron to depolarize. But the MHH model generates action potentials under the same conditions. The frequency of the sinusoidal stimulus affects the generation of the action potential. When the frequency is low, there is sufficient time to depolarize, and the action potential occurs (Figure 14). When *T*_*in*_ = 5 ms, the HH and MHH spiking models produce the action potentials, their spiking patterns belong to tonic spikes in pyramidal neurons. When *T*_*in*_ = 20 ms, the MHH model generates the repetitive bursts with doublet spikes, and the HH model performs the tonic spiking. When the value of *T*_*in*_ is increased to 60 ms, the action potential cannot be produced in the HH spiking model but can be obtained in the MHH model. The frequency range of the sinusoidal stimulus in the MHH spiking model is wider than that of the HH spiking model. The various spiking patterns can be obtained by appropriately adjusting the frequency of the sinusoidal signal.

## 6. Conclusion

The biological neuron is expressed adequately by the classic HH spiking model. It is sensitive to the temperature, the strength of the external stimulus, and the action time of the stimulus. The MHH spiking model successfully simulates the generation of the action potential in a neuron. When the different external stimuli are applied to the HH and MHH spiking models, the action potential is produced, and various spiking patterns are achieved. The MHH spiking model has advantages in generating the action potential through the comparison with the HH spiking model. The waveforms with smaller perturbations formed by the MHH spiking model are smooth. The higher frequency of the external stimulus, the more action potentials generated. The response speed of the MHH spiking model is faster than that of the HH spiking model. The various spiking behaviors are obtained by adjusting the signal frequency in the MHH spiking model. And meanwhile, the combination between neuron models and a memristor provides the possibility to scale down the neuron circuit and gives a novel way to replicate the functions of the biological neuron.

## Data Availability Statement

The original contributions generated for the study are included in the article/supplementary material, further inquiries can be directed to the corresponding author/s.

## Author Contributions

XF built models and simulations, carried out the experimental analysis, and prepared the manuscript in this work. SD and LW supervised the content of the article and the results of the simulations. All authors contributed to the article and approved the submitted version.

## Funding

Project supported by the National Key R and D Program of China (Grant No. 2018YFB1306600), the National Natural Science Foundation of China (Grant Nos. 62076207, 62076208, U20A20227), the Fundamental Science and Advanced Technology Research Foundation of Chongqing, China (Grant No. cstc2017jcyjBX0050).

## Conflict of Interest

The authors declare that the research was conducted in the absence of any commercial or financial relationships that could be construed as a potential conflict of interest.

## Publisher's Note

All claims expressed in this article are solely those of the authors and do not necessarily represent those of their affiliated organizations, or those of the publisher, the editors and the reviewers. Any product that may be evaluated in this article, or claim that may be made by its manufacturer, is not guaranteed or endorsed by the publisher.
